# Single sample scoring of molecular phenotypes

**DOI:** 10.1186/s12859-018-2435-4

**Published:** 2018-11-06

**Authors:** Momeneh Foroutan, Dharmesh D. Bhuva, Ruqian Lyu, Kristy Horan, Joseph Cursons, Melissa J. Davis

**Affiliations:** 1 University of Melbourne Department of Surgery, St. Vincent’s Hospital, Melbourne, VIC 3065 Australia; 2 Division of Bioinformatics, Walter and Eliza Hall Institute of Medical Research, Melbourne, VIC 3051 Australia; 3 School of Mathematics and Statistics, Faculty of Science, University of Melbourne, Melbourne, VIC 3010 Australia; 4 Department of Medical Biology, Faculty of Medical and Health Sciences, University of Melbourne, Parkville, VIC 3010 Australia; 5 Department of Biochemistry and Molecular Biology, Faculty of Medicine, Dentistry and Health Sciences, University of Melbourne, Melbourne, VIC 3010 Australia

**Keywords:** Single sample, Gene set score, *Singscore*, Gene signature, Gene set enrichment, Transcriptome, Molecular features, Molecular phenotypes, Dimensional reduction, Personalised medicine

## Abstract

**Background:**

Gene set scoring provides a useful approach for quantifying concordance between sample transcriptomes and selected molecular signatures. Most methods use information from all samples to score an individual sample, leading to unstable scores in small data sets and introducing biases from sample composition (e.g. varying numbers of samples for different cancer subtypes). To address these issues, we have developed a truly single sample scoring method, and associated *R/Bioconductor* package singscore (https://bioconductor.org/packages/singscore).

**Results:**

We use multiple cancer data sets to compare *singscore* against widely-used methods, including GSVA, *z*-score, PLAGE, and ssGSEA. Our approach does not depend upon background samples and scores are thus stable regardless of the composition and number of samples being scored. In contrast, scores obtained by GSVA, *z*-score, PLAGE and ssGSEA can be unstable when less data are available (*N*_*S*_ < 25). The *singscore* method performs as well as the best performing methods in terms of power, recall, false positive rate and computational time, and provides consistently high and balanced performance across all these criteria. To enhance the impact and utility of our method, we have also included a set of functions implementing visual analysis and diagnostics to support the exploration of molecular phenotypes in single samples and across populations of data.

**Conclusions:**

The *singscore* method described here functions independent of sample composition in gene expression data and thus it provides stable scores, which are particularly useful for small data sets or data integration. Singscore performs well across all performance criteria, and includes a suite of powerful visualization functions to assist in the interpretation of results. This method performs as well as or better than other scoring approaches in terms of its power to distinguish samples with distinct biology and its ability to call true differential gene sets between two conditions. These scores can be used for dimensional reduction of transcriptomic data and the phenotypic landscapes obtained by scoring samples against multiple molecular signatures may provide insights for sample stratification.

**Electronic supplementary material:**

The online version of this article (10.1186/s12859-018-2435-4) contains supplementary material, which is available to authorized users.

## Background

Several approaches have been developed to score individual samples against molecular signatures (or gene sets), including: ssGSEA (single sample gene set enrichment analysis) [1], GSVA (gene set variation analysis) [2], PLAGE (pathway level analysis of gene expression) [3] and combining *z*-scores [4].

Hänzelmann et al. (2013) implemented all four of these methods within the *R/Bioconductor* package GSVA and performed a detailed comparison [2, 5]. It should be noted that GSVA, PLAGE and *z*-scores use data from all samples in the very first step to estimate gene distributions; GSVA performs kernel density estimation of the expression profile for each gene across all samples, while PLAGE and z-scores perform standardisation. Although the ssGSEA implementation in the GSVA package normalises the scores across samples, this is the final step and it can be disabled. The ssGSEA method is also implemented through the GenePattern web-tool [6] which does not normalise scores by default. Some methods make assumptions about the data which may be unsuitable in certain cases, for instance, PLAGE and combined *z*-scores are parametric methods that assume normality of expression profiles, while the combined *z*-scores method additionally makes an independence assumption for genes in a gene set [7].

Here, we introduce a rank-based single sample scoring method, *singscore*. Using breast cancer data and several gene expression signatures we compare our approach to the methods listed above. The *singscore* method is simple, making the scores directly interpretable (as a normalised mean percentile rank), and our comparisons show that it is not only fast, but it also produces stable and reproducible scores regardless of the composition and number of samples within the data. Finally, we include examples from breast cancer gene expression data to show visualisation options and demonstrate the application of the *singscore* method for molecular phenotyping in a clinical context.

## Methods

### The singscore method

For a sample transcriptome which has been corrected for technical within-sample bias (i.e. RPKM, TPM, or RSEM data for RNA-seq after filtering for genes with low-counts), genes are ranked by increasing mRNA abundance.

For bidirectional gene signatures (with separate up- and down- regulated gene sets) or unidirectional signatures with known direction (e.g. all genes are up-regulated), genes are ranked based on their transcript abundance in increasing order for the up-set and in decreasing order for the down-set. Mean ranks are separately normalised relative to the theoretical minimum and maximum values, centered on zero and then summed to provide the score (i.e. ${\overline{S}}_{total,i}={\overline{S}}_{up,i}+{\overline{S}}_{down,i}$) which ranges between − 1 and 1. A sample with a high score can be interpreted as having a transcriptome which is concordant to the specified signature, and scores reflect the relative mean percentile rank of the target gene sets within each sample. The score (*S*) and normalised score ($\overline{S}$) are defined as:

1 $$ {S}_{dir,i}=\left(\frac{\sum_g{R}_{dir,i}^g}{N_{dir,i}}\right) $$

2 $$ {\overline{S}}_{dir,i}=\frac{\left({S}_{dir,i}-{S}_{\mathit{\min},i}\right)}{S_{\mathit{\max},i}-{S}_{\mathit{\min},i}} $$

Where:

- *dir* is the gene set direction (i.e. expected up- or down- regulated genes);
- *S*_*dir*, *i*_ is the score for sample *i* against the directed gene set;
- ${R}_{dir,i}^g$ is the rank of gene *g* in the directed gene set (increasing transcript abundance for expected up-regulated genes and decreasing abundance for expected down-regulated genes);
- *N*_*dir*, *i*_ is the number of genes in the expected up- or down-regulated gene set that are observed within the data (i.e. signature genes not present within the RNA abundance data are excluded);
- ${\overline{S}}_{dir,i}$ is the normalised score for sample *i* against genes in the signature, and;
- *S*_*min*, *i*_ and *S*_*max*, *i*_ are the theoretical minimum and maximum mean ranks which can be derived from an arithmetic sum (assuming unique ranks); for a series of *n* numbers starting at *a*_*1*_ and with a constant difference *d*, the sum can be calculated as $$ \left(\raisebox{1ex}{$n$}\!\left/ \!\raisebox{-1ex}{$2$}\right.\right)\left(2{a}_1+\left(n-1\right)d\right) $$. Setting *a*_*1*_ = 1, *d* = 1, *n* = *N*_*dir, i*_ and dividing through by *N*_*dir, i*_ to obtain the mean:

3 $$ {S}_{\mathit{\min},i}=\frac{\left({N}_{dir,i}+1\right)}{2} $$

Similarly, the maximum value can be obtained by setting *a*_*1*_ = (*N*_*total*_ – *N*_*dir*_):

4 $$ {S}_{\mathit{\max},i}=\frac{\left(2{N}_{total,i}-{N}_{dir,i}+1\right)}{2} $$

Where:

- *N*_*total*, *i*_ is the total number of genes in sample *i*.

For undirected gene signatures (without a known or expected direction for changes in expression), we compute the average, absolute, median-centred rank (Eqs. 5 & 6). As the direction of genes in the gene-set are unknown, this method only provides evidence of enrichment of more-extreme gene ranks in either direction, not necessarily information about the specific direction (Additional file 1: Figure S1). Resulting scores range between 0 and 1; as shown in Eqs. 7 & 8, the normalisation step is altered:

5 $$ {S}_i=\left(\frac{\sum_g{\widehat{R}}_i^g}{N_i}\right) $$

6 $$ {\overline{S}}_i=\frac{\left({S}_i-{S}_{\mathit{\min},i}\right)}{S_{\mathit{\max},i}-{S}_{\mathit{\min},i}} $$

Where:

- *S*_*i*_ is the score for sample *i* against the undirected gene-set;
- ${\widehat{R}}_i^g$ is the absolute, median-centered rank of gene *g* in the undirected gene set; i.e.
  ${\hat{R}}_i^g=\mid {R}_i^g-c\mathrm{e} il\left(\frac{N_{total}}{2}\right)\mid$, where *ceil* represents the ceiling function;
- *N*_*i*_ is the number of genes in the undirected gene set that are observed within the data (i.e. signature genes not present in the RNA abundance data are excluded);
- ${\overline{S}}_i$ is the normalised score for sample *i* against the undirected gene-set, and
- *S*_min, *i*_ and *S*_max, *i*_ are the theoretical minimum and maximum mean ranks obtained from the arithmetic series expansion. For undirected gene-sets, the minimum (*S*_min, *i*_) and maximum (*S*_max, *i*_) values can be derived as:

7 $$ {S}_{\min, i}=\frac{\left( ceil\left(\frac{N_{dir,i}}{2}\right)+1\right)}{2} $$

8 $$ {S}_{\max, i}=\frac{\left({N}_{total,i}- ceil\left(\frac{N_{dir,i}}{2}\right)+1\right)}{2} $$

As shown above, scores are normalized against theoretical minimum and maximum values, centered on zero (directed signatures) and if applicable (bidirectional signatures) scores are then summed. Centering and normalization is performed in this manner to maintain independence between samples. This is similar to a Wilcoxon rank-sum test when examining an expected up- or down-regulated gene set.

If users have a gene set associated with a specific cell phenotype and these genes undergo large changes in expression, for example, the results from a differential expression analysis where genes are filtered by significance (low FDR or adjusted *p*-value) and abundance (high mean logCPM or logTPM), then, *singscore* can score individual samples with an estimate of significance. Under the null hypothesis that members of the expected up-regulated genes are not enriched within high-abundance transcripts (and/or expected down-regulated genes are not enriched within low abundance transcripts), a permutation test with random gene sets can be performed. Care should be taken when interpreting such results without a specific biological hypothesis as gene sets expressed at high levels and/or heavily influenced by the experimental protocol (e.g. ribosomal gene RNAs) may lead to spurious conclusions. As shown in Additional file 1: Figure S2, when using a TGFβ-EMT gene set [8] to score a TGFβ treated sample, the score greatly exceeds most permuted scores, while scores of control samples appear near the lower tail of the null distribution. When an individual (e.g. patient) sample is scored with an appropriate signature, this can provide some confidence that the transcriptome is concordant with the gene set (which may be associated with response to specific therapies or drugs).

### Implementation of singscore

All statistical analyses were performed using *R* (v. 3.3 and greater) and *Bioconductor* (v. 3.4 and greater). We have produced an *R/Bioconductor* package, singscore, to implement this method, and have included several visualisation functions that produce both static (using ggplot2 [9]) and interactive (*.html;* using plotly [10]) plots.

### Other scoring methods

The *R/Bioconductor* package GSVA (v1.26.0) was used to evaluate the performance of the GSVA, ssGSEA, z-score and PLAGE methods [7]. We have modified this approach slightly to account for bidirectional signatures where both expected up- and down-regulated gene sets were available, with a method previously described in Foroutan et al. [8]. As the ssGSEA method implemented by GenePattern [6] does not normalise scores, we have also included ssGSEA_!Norm_, by removing the (final) normalisation step in the GSVA package implementation of ssGSEA, to test performance in smaller data sets with less samples. However, it should be noted that while ssGSEA scores obtained from the GSVA package and GenePattern are highly correlated, the scores are not directly comparable (Additional file 1: Figure S3).

### Data

In this study, we used The Cancer Genome Atlas (TCGA) breast cancer [11] RNA-seq data (RSEM normalised) and microarray data (RMA normalised from Agilent4502A_07_03 microarray platform), the Cancer Cell Line Encyclopaedia (CCLE) [12] breast cancer cell line RNA-seq data (TPM normalised), raw fastq files for breast cancer cell lines from Daemen et al. [13] (re-calculated as RPKM; *see* *Data processing* *below*), and the integrated cell line TGFβ-EMT data from Foroutan et al. [8] (Table 1).

**Table 1:** List of data sets used in the current study

Data	Source	Date accessed	Reference
TCGA RNA-seq	The UCSC Cancer Genomics Browser [30]	February 2016	PMID: 23000897
TCGA microarray	The UCSC Cancer Genomics Browser [30]	October 2015	PMID: 23000897
CCLE RNA-seq	Cancer Cell Line Data Repository [31]	April 2017	PMID: 22460905
Daemen et al. RNA-seq	Gene Expression Omnibus [32] ID: GSE48213	July 2016	PMID: 24176112
TGF*β*-EMT	Data [33] from Foroutan et al. [8]	September 2017	PMID: 28119430
GSE79235	Gene Expression Omnibus [32]	April 2018	PMID: 27154822

### Data processing

The SRA files from Daemen et al. were obtained July 2016 (GSE48213), and converted to fastq files using the fastq-dump function in the SRA toolkit [14]. Reads were aligned to the reference human genome hg19 using the Rsubread package [15] in *R/Bioconductor*, and count level data were obtained using the featureCount function with default parameters. The edgeR package [16] was used to calculate RPKM values. All RNA-seq data were filtered to remove genes with low counts in most samples; for example, for TCGA breast cancer data, genes were retained if they had RSEM abundance > 2 in more than 90% of samples. For all other data, we used processed versions available online (Table 1).

### Simulations for comparing methods

#### Stability

Method stability was examined using 500 TCGA breast cancer samples with both RNA-seq and microarray data (Sample IDs in Additional file 2: Table S1), sub-sampled to vary the number of samples and genes present for each evaluation. To examine sample size effects upon a given sample, *s*_*i*_, two data sets were created by sampling from both the RNA-seq and microarray data to select a sample *s*_*i*_ and *n* − 1 other random samples. The score for sample *s*_*i*_ was then computed using all listed methods, and this process was repeated across all 500 samples at a given sample size, such that there are 500 matched scores in total from both the microarray data and RNA-seq data. The Spearman’s rank correlation coefficient and concordance index were then calculated between sample scores from the microarray and the RNA-seq data. We note that for some methods sampling data in this manner can modify the background samples for a sample of interest, reflecting the influence of overall sample composition on the final scores. A similar analysis was performed by varying the number of genes, sub-sampling genes from the gene set of interest.

We performed this analysis with both epithelial and mesenchymal gene sets (expected up-regulated gene sets) [17], and the bidirectional TGFβ-EMT signature [8], varying the number of samples, *N*_*S*_ = (2, 5, 25, 50, 500), and genes, *N*_*G*_ = (1000, 3000, 5000, 10000, *ALLGENES*). All permutations were repeated 20 times to estimate error margins.

#### Power analysis and type 1 error

We evaluated the power of each method to differentiate biologically distinct groups. For this, we simulated RNA-seq data using methods from Law et al. [18]. An inverse chi-squared distribution was used to model dispersion and the library size was left constant at 1.1 × 10^7^. We simulated *N*_*S*_ = 30 samples and *N*_*G*_ = 1000 genes, representing two biological conditions (*N*_*S*_ = 15 in each group) with 30 differentially expressed genes (DEGs) between them. This simulation was repeated 100 times, each time creating three gene sets of size 30 to represent the three scenarios with different signal to noise ratios: (i) when 50% of the genes in the gene set were differentially expressed (15 DEGs and 15 non-DEGs), (ii) when 80% of the genes were differentially expressed (24 DEGs and 6 non-DEGs), and (iii) when none of the genes were differentially expressed (30 non-DEGs). We also varied the logFC of DEGs (effect size) across the two conditions. We then used all methods to score samples against these gene sets and applied a *t*-test on the scores to evaluate the performance at separating the two conditions. The statistical power and type 1 error (false positive rate) were estimated at *α* = 0.05 for each effect size with a given signal to noise ratio. The power was calculated as the proportion of the positive tests (*p*-value < 0.05) in 100 simulations for each scenario (50% DE and 80% DE). The type 1 error was calculated as the proportion of simulations where non-DEG sets tested positive (i.e. false positives).

#### Gene-set recall

Next we compared methods for their ability to produce different scores for two conditions with differentially expressed gene sets. RNA-seq data were again simulated using the method of Law et al. [18] with *N*_*S*_ = 30 and *N*_*G*_ = 10,000, representing two biological conditions (*N*_*S*_ = 15 in each group) with 2000 DEGs (logFC or effect size = 1.1). We repeated this simulation 100 times, each time creating (i) 500 gene sets (of size 30) where 50% of genes were DE, (ii) 500 gene sets where 80% of genes were DE, and (iii) 500 gene sets where genes were randomly sampled, representing gene sets with no signal. We then scored samples against all 1500 gene sets in each simulation and performed a *t*-test between the group scores. Next, *p*-values from the 50% DEG set (500 *p*-values) and non-DEG set (500 *p*-values) were combined and adjusted for multiple hypothesis testing to produce estimated *q*-values [19]. These *q*-values were thresholded at FDR = 0.05, with gene sets that tested positive considered as DEG sets. Performance of these predictions was quantified using the F1 score which accounts for both the precision and recall of each method. This was repeated for *p*-values from the 80% DEG sets and non-DEG sets.

### Comparing the computation time for scoring methods

To compare the computational time of each scoring method, we randomly selected 10,000 gene sets from MSigDB signatures [20, 21] and all methods were used to score subsets of the TCGA breast cancer RNA-seq data with either 25 samples or 500 samples. This was repeated 20 times to improve coverage of signatures on MSigDB and allow variance estimates for the computation times. This comparison was performed on a UNIX machine (Intel(R) Xeon ® CPU E5–2690 v3 @ 2.60GHz) without code parallelisation.

## Results

### Technical considerations for singscore

#### *Singscore* results are highly stable compared to other scoring approaches

**Fig. 1:**
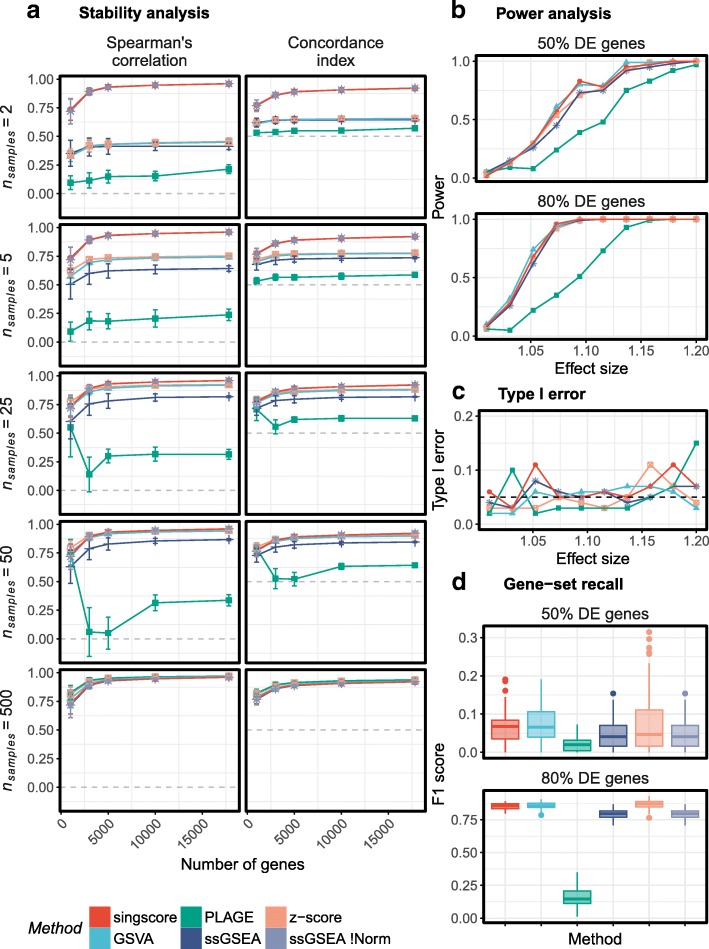
**a** Comparing the stability of scoring methods to changes in the number of samples and genes within transcriptomic data. For both Spearman’s correlation coefficients and concordance index, a higher value indicates better performance, with 0 and 0.5, respectively, indicating poor performance for each method. Similar results were observed when other signatures were used for scoring (Additional file 1: Figure S4 and S5); **b** Comparing the power of methods to distinguish groups with distinct biology; **c** Comparing the type 1 error for different methods when distinguishing groups with distinct biology; **d** Comparing the ability of methods to call true differential gene sets between two conditions

Performance of the *singscore* method was compared to GSVA, z-score, PLAGE, ssGSEA, and ssGSEA without normalization (ssGSEA_!Norm_), using both microarray and RNA-seq data from the TCGA breast cancer cohort. Overlapping samples between the two platforms (*N*_*S*_ = 500) were scored using three gene signatures: the epithelial, mesenchymal, and TGFβ-induced EMT (TGFβ-EMT) signatures [17], while the number of samples and genes in the data were varied (details given in *Methods*). The Spearman’s correlation and concordance index [22] between sample scores from the two platforms were calculated. Our results show good stability for *singscore* and ssGSEA_!Norm_ compared to the other methods when varying the sample number and number of genes in the data (Fig. 1a, and Additional file 1: Figures S4 & S5), reflecting sample composition effects. While all methods performed well for large data sets, PLAGE had the worst performance with sub-sampled data, whereas GSVA, z-score and ssGSEA showed a reduced stability compared to the *singscore* and ssGSEA_!Norm_ in data sets with small sample sizes (*N*_*S*_ < 25). This demonstrates that the *singscore* may be particularly useful in cases where sample numbers are relatively low, or where there may be a heterogeneous sample composition (i.e. samples across different cancer subtypes with unbalanced frequencies). We depict these effects by changing the balance/composition of samples under two settings: (1) overlaying mesenchymal scores for control and TGFβ-treated cell lines (*N*_*S*_ = 2–4) on to the score distributions for a larger set of samples related to these groups (*N*_*S*_ = 55–57; Additional file 1: Figure S6), and (2) assessing the stability of scores in data with a small number of control and TGFβ-treated cell lines (*N*_*S*_ = 2–4; Additional file 1: Figure S7). Although PLAGE appeared to perform poorly in many comparisons performed here, we believe this may reflect the fact that the underlying metric was not designed to account for directionality as discussed below.

#### *Singscore* has high power and gene-set recall ability

Using two simulation settings (see *Methods*) we compared *singscore* to other approaches, assessing each method’s power to distinguish biologically-distinct sample groups, and each method’s ability to call differential gene sets between two groups. The power analysis (Fig. 1b) showed that with the exception of PLAGE, all methods performed equally well, and had similar false positive rates or type 1 errors (Fig. 1c). Examining gene-set recall (Fig. 1d), the *singscore*, GSVA and z-score methods performed best when 80% of genes were differentially expressed. All methods had relatively poor performance when only 50% of gene set genes were differentially expressed, however, *singscore* and GSVA had slightly higher F1 scores.

#### The *singscore* method is computationally fast

An important factor for computational tools is run-time and we note that ssGSEA_!Norm_ and ssGSEA have much longer compute times than all other methods when tested with random signatures from MSigDB [20, 21] (Additional file 1: Figure S8; details in *Methods*), whereas *singscore* is very fast and comparable with GSVA, PLAGE and z-score*.*

### Application of singscore

#### Obtaining landscapes of molecular phenotypes

**Fig. 2:**
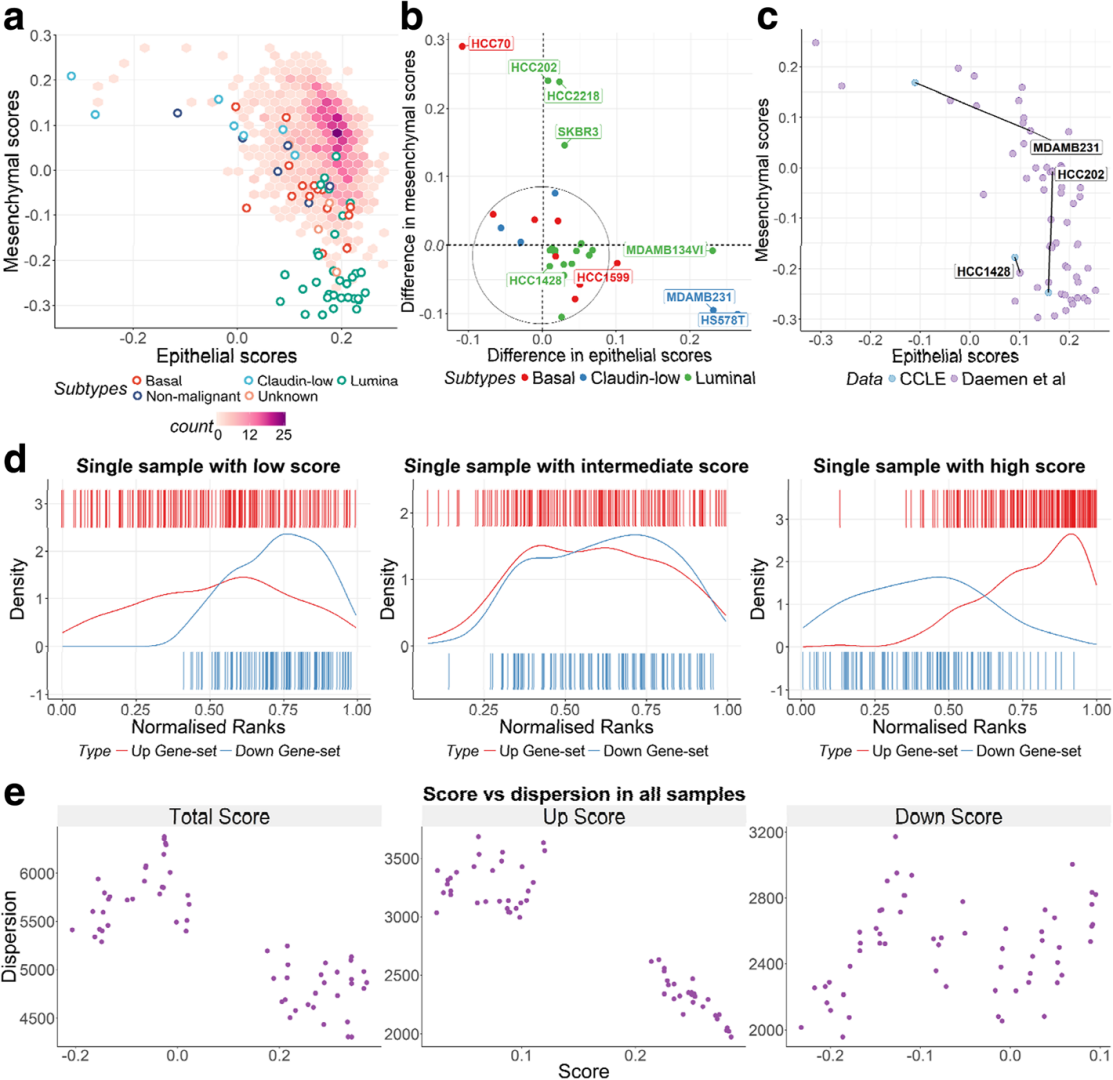
**a** Epithelial and mesenchymal scores obtained from *singscore* for the TCGA breast cancer samples (*hexbin density plot*) and a collection of breast cancer cell lines (*circle markers, coloured by subtype*). Note that as per the original study by Tan et al., the epithelial and mesenchymal signatures are distinct (but overlapping) for tumours and cell lines; **b** Differences in epithelial and mesenchymal scores for 32 overlapping breast cancer cell lines between Daemen et al. and the CCLE datasets. The majority of cell lines show relatively consistent scores in these two data sets (circled in the lower left corner); **c** The HCC1428 cell line has very similar scores in each dataset, while the MDA-MB-231 cell line has a large shift in epithelial score, and the HCC202 cell line has a large shift in mesenchymal score; **d** Three microarray samples from the TGFβ- EMT data set [8] with low, medium and high scores for the TGFβ-EMT signature; **e** Scatter plots demonstrating the relationship between rank dispersions (MAD) and scores obtained by *singscore,* for: total score (combined up- and down-set scores), distinct expected up-regulated gene set scores, and distinct expected down-regulated gene set scores

Scoring samples against multiple molecular signatures and plotting them in 2D can be useful to stratify samples based on the associated molecular phenotypes of samples. For example, scoring TCGA breast cancer samples (*N*_*S*_ = 1091 RNA-seq) and a collection of breast cancer cell lines [13] (*N*_*S*_ = 64 RNA-seq) against mesenchymal and epithelial signatures from Tan et al. [17] (tumour and cell line signatures, respectively) can refine the stratification of patients and cell lines. Figure 2a shows that samples with a high mesenchymal and low epithelial score across the independent data sets [23] are particularly enriched for a subset of aggressive, claudin-low breast cancers. These samples have different expression profiles when compared to samples with high epithelial and low mesenchymal scores, enriched for a subset of samples from a less aggressive subtype (e.g. luminal-A/B tumours and luminal cell lines). Each sub-group can be further analysed and contrasted, for example by comparing different -omics data across these sub-groups, or by examining their associations with patient survival or cell line drug response.

#### Comparing consistency of scores from independent data sets

As *singscore* does not depend on the composition nor size of a data set, it is tempting to speculate that cell line scores for a given signature will be consistent across independent data sets. To examine this, we compared transcriptomic data for breast cancer cell lines collected from two studies [12, 13], and calculated both the epithelial and mesenchymal scores across 32 overlapping cell lines. As shown, the scores are largely consistent (Fig. 2b), despite differences in computational pipelines, gene expression metrics and experimental protocols for the two datasets. For the small number of cell lines with substantial variation in scores, we cannot exclude the possibility that variation between the independently cultured cells (i.e. cell line drift) may underpin observed differences. We note that most cell lines with a large variation in mesenchymal scores are from luminal sub-groups with consistently high epithelial scores across the two datasets, while cell lines with the highest variability in epithelial scores are from the claudin-low sub-groups which also show consistently high mesenchymal scores (Fig. 2b). Variation in epithelial and mesenchymal scores for three representative cell lines (HCC1428, HCC202, and MDAMB231) is illustrated in Fig. 2c.

#### Assessment of scores: beyond a single value

When genes are ranked by increasing abundance, higher ranks for expected up-regulated genes, and lower ranks for expected down-regulated genes indicate that the samples transcriptional profile is concordant with the sample gene set being scored. Considering gene set ranks which approach the maximum theoretical mean-ranks within a sample (see Methods), the expected up- and down-regulated gene sets should form an approximately bimodal distribution, with higher ranks for the up- and lower ranks for the down-set. For samples that are not strongly concordant with a signature, the distributions of genes are less coordinated, and often uniformly distributed over a wide range of values.

To easily visualise the rank distribution of genes in the up- and down-gene sets, the singscore *R/Bioconductor* package provides static and interactive plots that display density and barcode plots for gene ranks in individual samples (Fig. 2d). These plots help to interpret the score in the context of the ranked genes, and often demonstrate that up- and down- gene sets can vary in their dispersion, contributing to the range of ranks we observe. We often see that samples with a low score may have an inverted pattern of expression (Fig. 2d, panel at left), those with a score near the centre of the null distribution have no enrichment for the gene set (i.e. randomly distributed gene ranks; Fig. 2d, middle panel), while high scoring samples are concordant with the gene set tested (Fig. 2d, panel at right).

To illustrate these differences, we alo calculate median absolute deviation (MAD) of the gene set ranks to estimate relative rank dispersion. Plotting scores against dispersion for the samples in the TGFβ-EMT data shows that samples with a high total score also have lower dispersion, demonstrating more coordinated changes in the up- and/or down-sets in these samples (Fig. 2e). The less obvious yet useful application of this statistic is differentiating samples with similar scores but distinct dispersion profiles; for example two samples may have a similar gene set mean-rank, but in one sample these may be tightly clustered and in the other they may be uniformly distributed across a much larger range, reflecting different regulation of this gene set. It is also possible to look at the rank dispersion of the up- and down- sets to assess the performance of each one separately. Fig. 2e shows that genes in the expected up-regulated set of TGFβ-EMT signature are more coordinated in samples compared to the down-set. These visualisations may be used as diagnostic tools to help users interpret gene set scores and possibly improve them by identifying and filtering out less informative genes.

## Discussion

We have described a rank-based single sample gene set scoring method, implemented in the *R/Bioconductor* package singscore. Our method can easily be applied on any high throughput transcriptional data from microarray or RNA-seq experiments. While our method is non-parametric, genes with low read counts should be filtered out, adjusted for gene length [24, 25], and ideally for GC content bias [26] and other technical artefacts [27, 28], because these may alter gene ranks within individual samples. Accordingly, RNA abundance data formatted as RPKM, TPM, or RSEM can be used, with or without log-transformation.

Using microarray and RNA-seq platforms of the TCGA breast cancer data, we show that our *singscore* approach yields stable scores for individual samples because they are treated independently from other samples, in contrast to GSVA, PLAGE, z-score, and ssGSEA (Fig. 1a). Although modifying ssGSEA to exclude the final normalization step (ssGSEA_!Norm_) also results in stable scores, the raw scores produced by the ssGSEA_!Norm_ algorithm cannot be directly interpreted (e.g. a value of 0 carries no context). This issue became apparent when comparing unnormalized ssGSEA scores from either the GSVA or GenePattern implementations (using the same parameters) where it was observed that while the scores are highly correlated they are not directly comparable (Additional file 1: Figure S3). While normalisation procedures used by GSVA and ssGSEA can be useful with large representative data sets, scoring data subsets where the relative composition of sample types varies (such as can occur with permutation testing) can cause the score of an individual sample to be unstable. Evaluation of the type of small, imbalanced dataset which may be encountered in a clinical context is shown in Additional file 1: Figures S6 and S7. The *singscore* method includes per sample normalisation and scaling by considering the theoretical minima and maxima for scores in each sample, and can be applied to a single sample in isolation. We further show that this method has a high power to distinguish samples with distinct biology which is comparable to other methods, as well as high F1 scores when identifying gene sets with differential expression, similar to the GSVA and z-score methods (Fig. 1b, c and d).

We show that current implementations of both ssGSEA_!Norm_ and ssGSEA through the GSVA package are computationally much slower than all other methods when scoring samples against a large number of random signatures (Additional file 1: Figure S8), while our approach is fast. We note that while the performance of PLAGE is poor across the majority of comparisons performed in this study, this may be attributed to the fact that the activity of a gene set is computed by projecting samples onto the first eigen-vector of the expression matrix. Due to this computation, scores vary a lot with changes to sample composition, and the values may rotate around 0, similar to how projections of observations can vary when performing PCA on sub-sampled datasets. These observations suggest that the PLAGE method is fundamentally different from all the other scoring approaches and should be used only for within dataset analysis and not analyses between data sets.

We compared breast cancer cell lines overlapping between the CCLE and Daemen et al. data and showed high consistency in epithelial and mesenchymal scores obtained by our scoring approach for the majority of cell lines (Fig. 2b). Because only a small subset of cell lines show large differences in epithelial or mesenchymal scores between the two data sets it is tempting to speculate that variations in scores are not due to the differences in technical or computational pipelines, which would have affected all the cell lines in the analysis. Rather it is possible that differences reflect real biological variation in the molecular phenotypes of some cells: the more-variable cell lines within the Daemen et al. data have hybrid epithelial-mesenchymal phenotypes (i.e. high epithelial and mesenchymal scores); these cell lines may have a greater degree of epithelial-mesenchymal plasticity allowing variations in their EMT phenotype under different experimental conditions. Interestingly, all cell lines with relatively large variations along the mesenchymal axis and smaller differences in epithelial scores are luminal cell lines which in general are shown to have strong epithelial phenotype (Fig. 2b, and [23]), while most cell lines with large shifts on the epithelial axis and little change in mesenchymal scores are claudin-low cell lines which have been shown to be strongly mesenchymal (Fig. 2b, and [23]).

More recent single sample methods such as personalized pathway alteration analysis (PerPAS) [29] have not been discussed here, as they are fundamentally different from our approach. For example, PerPAS needs topological information for each gene set to perform pathway analysis, and further uses either a control sample or a cohort of normal samples based on which the gene expression data in single samples are normalised [29]; these requirements make this method unsuitable for many available datasets.

We also note that methods requiring a large number of samples and a balanced composition to calculate a precise and stable score for individual samples may need to be re-run several times across a large data set when new samples are added. This adds extra complexity which may not be obvious to most users running such scoring methods. Our *singscore* method provides a simple and easy-to-understand pipeline which is also computationally fast. This method performs as well as the comparable scoring methods in large data sets in terms of stability, while outperforming them in smaller data sets by providing more stable scores, which are also easily interpretable. We further show that, excluding PLAGE which had relatively poor performance in these tests, all methods had a similar power, type I error, and/or F1 score when the signal to noise ratio was high, however, the *singscore*, GSVA and z-score performed slightly better for data with a less prominent signal. Several visualisation options at both the bulk and single sample level are provided in the *R/Bioconductor* package singscore to enable users to explore genes, gene signatures, and samples in more depth.

## Conclusion

In the context of personalised medicine there is an increasing need to examine data from an individual patient, or from a small number of samples in pre-clinical experiments. Current scoring methods are parametric and/or depend on a large number of samples to produce stable scores, while *singscore* generates scores that are stable across a range of sample sizes and numbers of measured genes. This is due to it being a non-parametric, rank-based, and truly single sample method. Moreover, scores generated by our method show high level of consistency across independent data sets and can be normalised at a single sample level, resulting in easily-interpretable scores.

## Additional files

Additional file 1: This includes eight supplementary figures supporting the conclusions of the article. (DOCX 1948 kb)

Additional file 2: This includes IDs for 500 of the overlapping samples between the TCGA breast cancer RNA-seq and microarray data. (TXT 8 kb)

## Abbreviations

CCLE: Cancer cell line encyclopaedia
EMT: Epithelial-mesenchymal transition
GSVA: Gene set variation analysis
PerPAS: Personalized pathway alteration analysis
PLAGE: Pathway-level activity of gene expression
ssGSEA: Single sample gene set enrichment analysis
TGFβ: Transforming growth factor beta
