# Supplementary material for: Single sample scoring of molecular phenotypes
Source: BMC Bioinformatics. 2018 Nov 6;19:404. doi: 10.1186/s12859-018-2435-4 (PMC6219008; doi:10.1186/s12859-018-2435-4)
Supplement: Supplementary file 1 — This includes eight supplementary figures supporting the conclusions of the article. (DOCX 1948 kb) [file 12859_2018_2435_MOESM1_ESM.docx]

**Supplementary results for: “Single sample scoring of molecular phenotypes”**

Momeneh Foroutan†^1,2^, Dharmesh D. Bhuva†^2,3^, Ruqian Lyu^2^, Kristy Horan^2^,
Joseph Cursons*^2,4^, Melissa J. Davis*^2,4,5^

^1^ University of Melbourne Department of Surgery, St. Vincent’s Hospital, Melbourne, VIC 3065, Australia.

^2^ Division of Bioinformatics, Walter and Eliza Hall Institute of Medical Research, Melbourne, VIC 3051, Australia.

^3^ School of Mathematics and Statistics, Faculty of Science, University of Melbourne, VIC 3010, Australia.

^4^ Department of Medical Biology, Faculty of Medical and Health Sciences, University of Melbourne, Parkville, VIC 3010, Australia

^5^ Department of Biochemistry and Molecular Biology, Faculty of Medicine, Dentistry and Health, University of Melbourne, VIC 3010, Australia.

# Comparing scores from directed and undirected gene sets

When dealing with directional gene sets (*e.g*. Up and Down), a sample where gene ranks indicate the opposite behaviour (*ie.* Up genes are lowly ranked and Down genes are highly ranked) will have a lower score than a sample where genes behave as expected. For undirected gene sets (*ie.* unknown direction), the scores will be high as long as the gene ranks are at either extreme. Accordingly, lower scores are associated with samples that have a random or centred distribution of signature gene ranks.


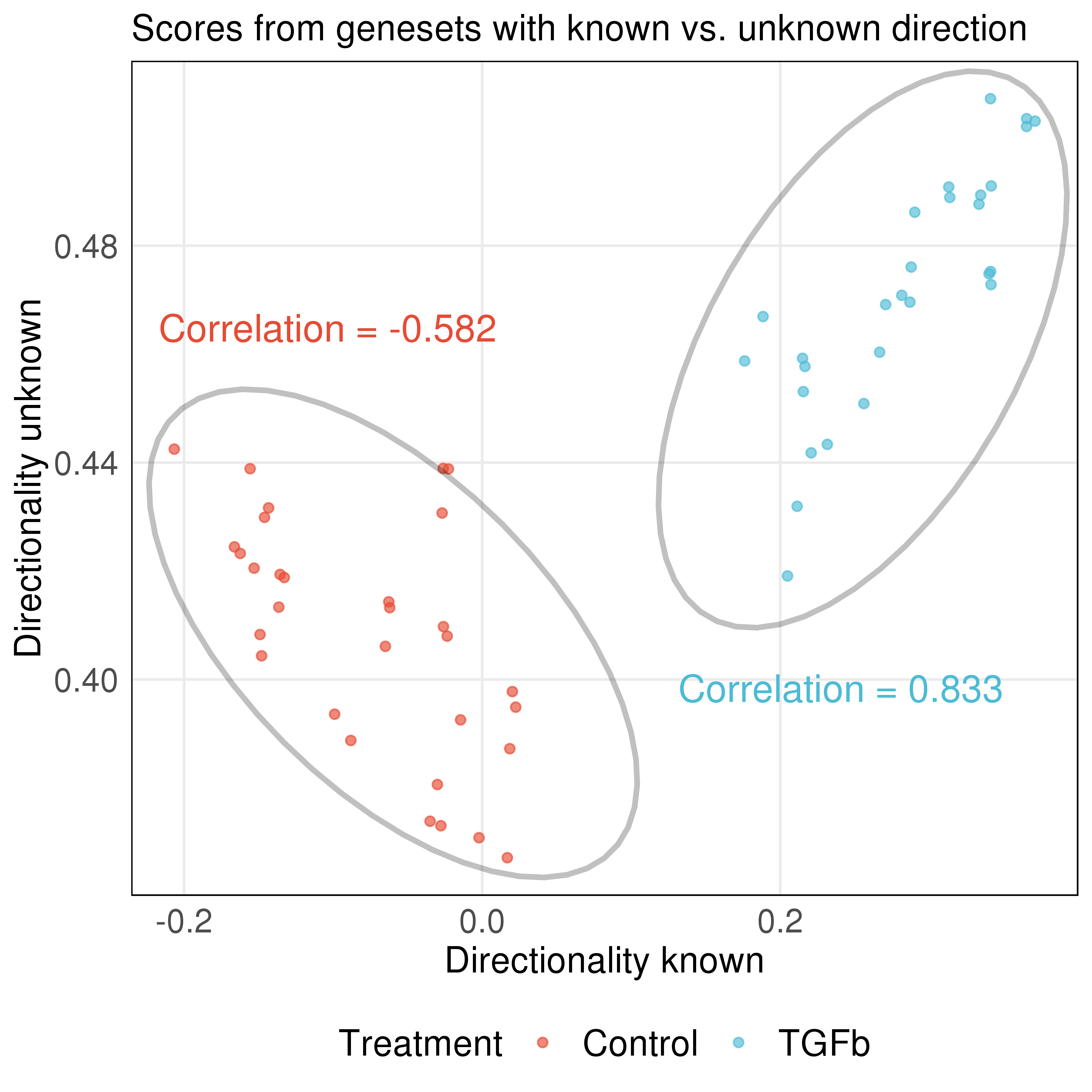


**Figure S1.** A set of control and TGFβ treated samples were scored with the TGFβ EMT signature using two different modes: On the *x* axis, we score samples with directional gene sets, while on the *y* axis we scored samples with the same gene set but with no directionality assumed. Comparing the scores, there is a strong positive correlation between directed and undirected signature scores for the TGFβ-treated samples. For the control samples however, low directed scores indicate that the genes are behaving in an opposite manner to that captured in the directed set but as expected show a negative correlation against the scores of the undirected list, showing that there is enrichment at more extreme ranks, even if much of it is in the opposite direction.

# Null distributions for gene-set scores within single samples

For each individual sample scored against a given signature, we provide functions that test the null hypothesis that the expected up-regulated genes are not enriched within high‑abundance transcripts (and/or expected down-regulated genes are not enriched within low abundance transcripts). The alternative hypothesis is assessed using a permutation test with random gene sets.


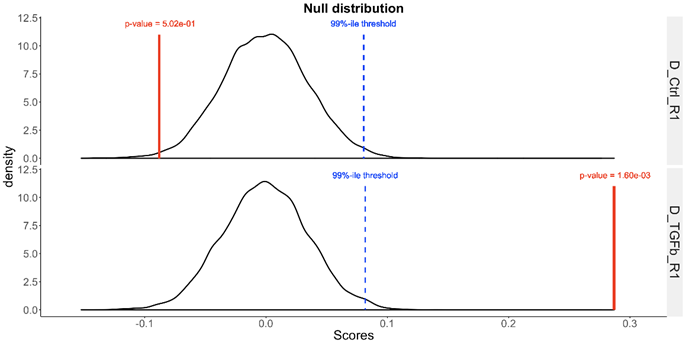


**Figure S2.** Null distributions for two samples from a TGFβ-EMT data set (*top*: control sample, *bottom*: TGFβ-treated sample) scored against the Foroutan (2017) TGFβ-EMT signature. Null distributions were generated with 1000 permutations, randomly sampling gene sets of the same size. Solid red lines indicate the observed scores and their associated *p*-values, and dashed blue lines represent significance with an empirical *p*-value < 0.01.

# Comparing ssGSEA scores from GenePattern and GSVA package

When comparing the GSVA (v. 1.26.0) and *GenePattern* (v. 9.0.9) implementations of ssGSEA we used identical settings (absolute ranking, weighting = 0.25 and no normalisation), however, as shown (Fig. S3), while the scores are highly concordant, there is an apparent offset and skew; because of the very large size of the scores, even these small deviations can result in very different scores that are difficult to directly compare (*e.g*.a sample may score 0 using one method and have a score of -1000 with the other).


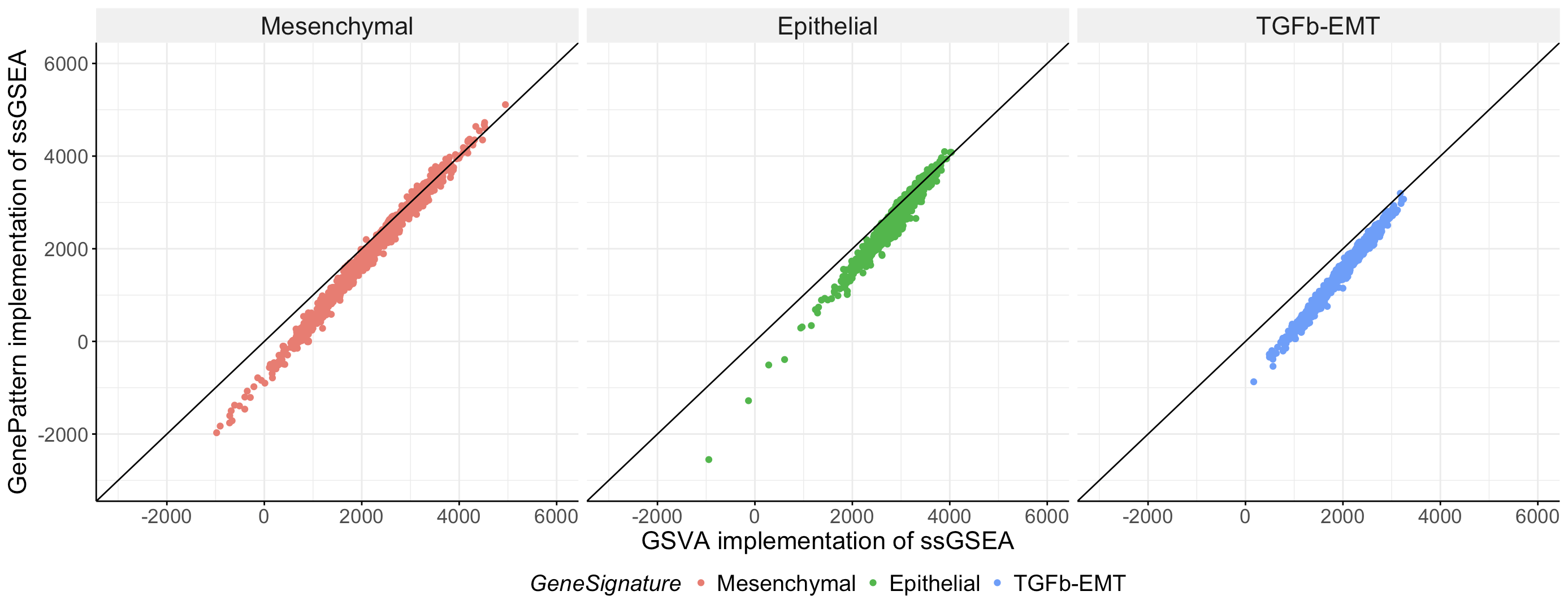


**Figure S3**. Comparisons between mesenchymal, epithelial and TGFβ-EMT ssGSEA scores obtained from GSVA package (*x* axis) and GenePattern (*y* axis). The same parameters were used for both implementations. The solid line shows the *x* = *y*.

# Stability of scores obtained from different methods

Simulation settings to assess scores stability are given in the main text (*Methods/Simulations for comparing methods*). Figures S4 and S5 show comparisons between score stability using different methods with epithelial and TGFβ-EMT signatures.

**
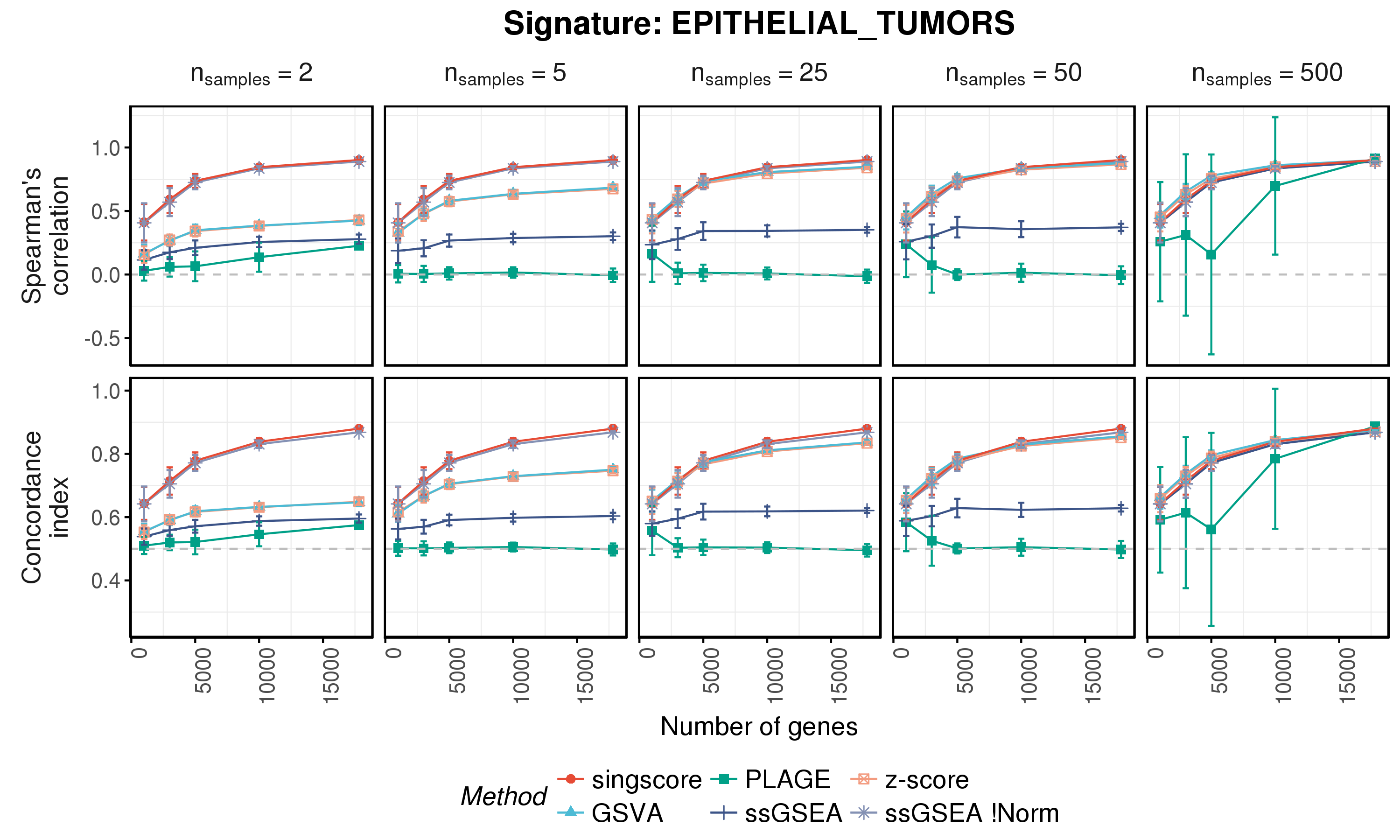
**

**Figure S4.** Comparing the stability of scoring methods with changes in number of samples and genes for transcriptomic data sets, using the epithelial signature from Tan *et al.* (2014). For both Spearman’s correlation and concordance index (C-Index) higher values indicate better performance, with 0 and 0.5 indicating poor performance for each method, respectively.

**
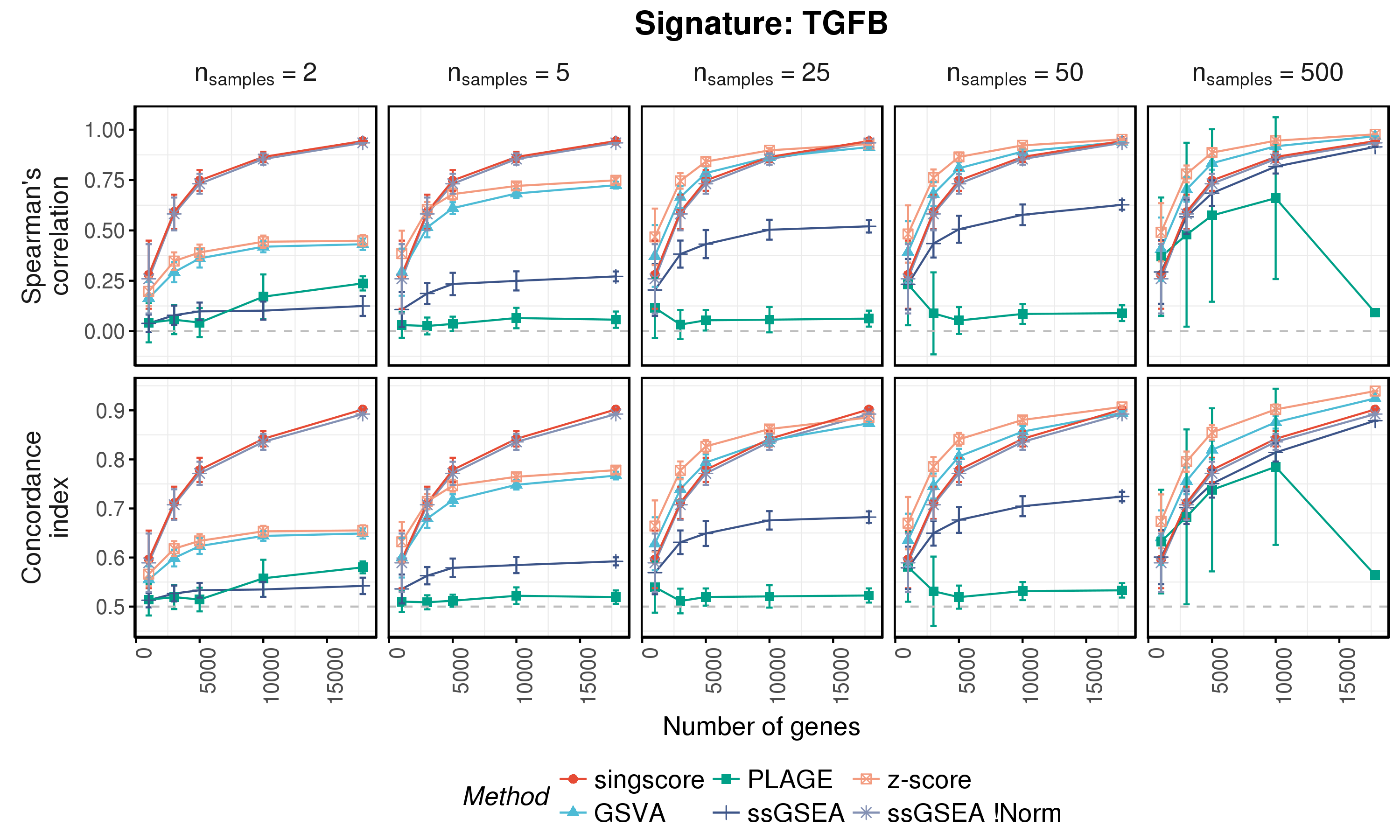
**

**Figure S5.** Comparing the stability of scoring methods with changes in number of samples and genes for transcriptomic data sets, using the TGFβ-EMT signature from Foroutan *et al.* (2017; with both up- and down- regulated gene sets). For both Spearman’s correlation and concordance index (C-Index) higher values indicate better performance, with 0 and 0.5 indicating poor performance for each method, respectively.

## Importance of score stability

To demonstrate the importance of score stability for different methods, we performed two separate analyses:

1. Using integrated TGFβ-EMT data, we retained a couple of samples and scored the remainder against epithelial and mesenchymal signatures, before scoring the hold-out samples separately and superimposing these on top of the other sample scores. A method with a higher stability will associate retained samples with the correct treatment group (TGFβ or control) regardless of the cell lines compositions. We performed this analysis with 5 scenarios that cover different sample compositions:
2. Only control cell lines (*N_Samples_* = 2)
3. Only TGFβ-treated cell lines (*N_Samples_* = 2)
4. 2 control and 1 TGFβ-treated cell lines (*N_Samples_* = 3)
5. 1 control and 2 TGFβ-treated cell lines (*N_Samples_* = 3)
6. 2 control and 2 TGFβ-treated cell lines (*N_Samples_* = 3)

Figure S6 demonstrates the scores for each of these scenarios. As shown, the *singscore* and ssGSEA_!Norm_ methods produce scores for new samples that align with the correct treatment group in all scenarios. The ssGSEA method shows a shift in scores, generating higher scores for retained samples. When samples from only one treatment group are compared (scenarios a and b) the z-score does not overlay samples with the correct categories, and GSVA and PLAGE produce large differences in scores which may be misleading. These two methods also perform poorly when the sample composition is imbalanced (scenarios c and d).

Accordingly, the stability is important to allow the comparison of scores when examining gene signatures with a known biological phenotype. The effects of composition are particularly important if users perform cross-validation with data partitioning, as scores obtained by other methods which vary with sample compositions are not comparable across different partitions/data folds.


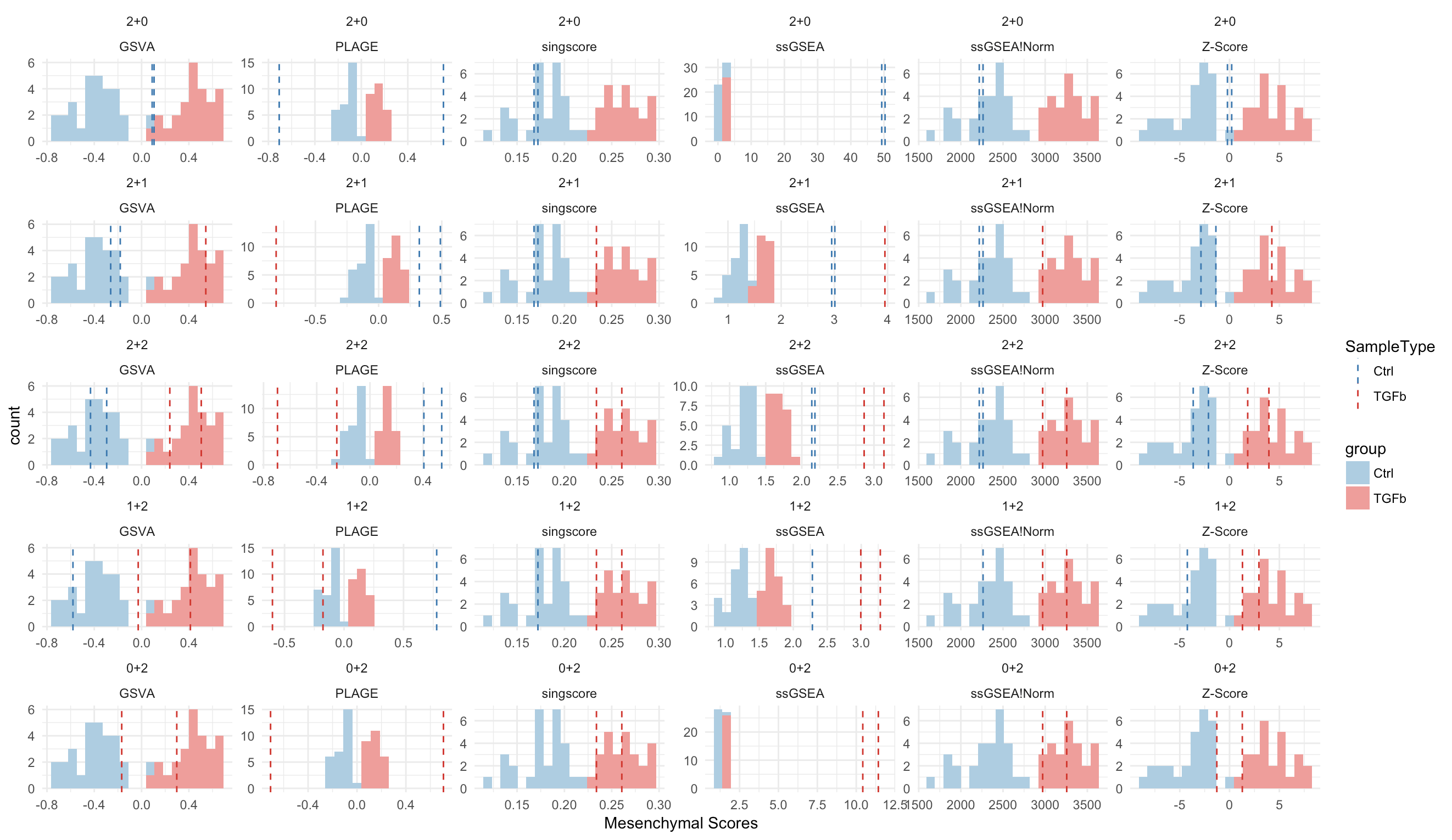


**Figure S6**. Histograms of mesenchymal scores obtained by different methods for the 55-57 samples which representing two treatment groups: control and TGFβ-treated. Dashed lines depict sample (control and/or TGFβ-treated) scores for the mesenchymal signature from Tan *et al.* (2014). Numbers in plot titles demonstrate the number of control and TGFβ-treated cell lines that were scored, respectively *(i.e*. 2+0: 2 control and 0 TGFβ-treated, 2+1: 2 control and 1 TGFβ-treated, etc). Data used for this analysis were downloaded from <https://doi.org/10.4225/49/5a2a11fa43fe3>.

1. In clinical settings when considering personalised treatments there may only be a small number of samples to analyse and it is important that the score obtained for an individual sample is consistent. To show the importance of score stability in small cohorts with different sample compositions, we use data (from GSE79235) for four samples: two control and two TGFβ-treated. Considering the five scenarios above, we scored samples using all methods. Figure S7 shows how scores for the same sample changes depending upon the other samples present. While *singscore* and ssGSEA_!Norm_ perform well in separating TGFβ-treated and control cell lines, scores from all the other methods change substantially with the number and composition of samples in the data.


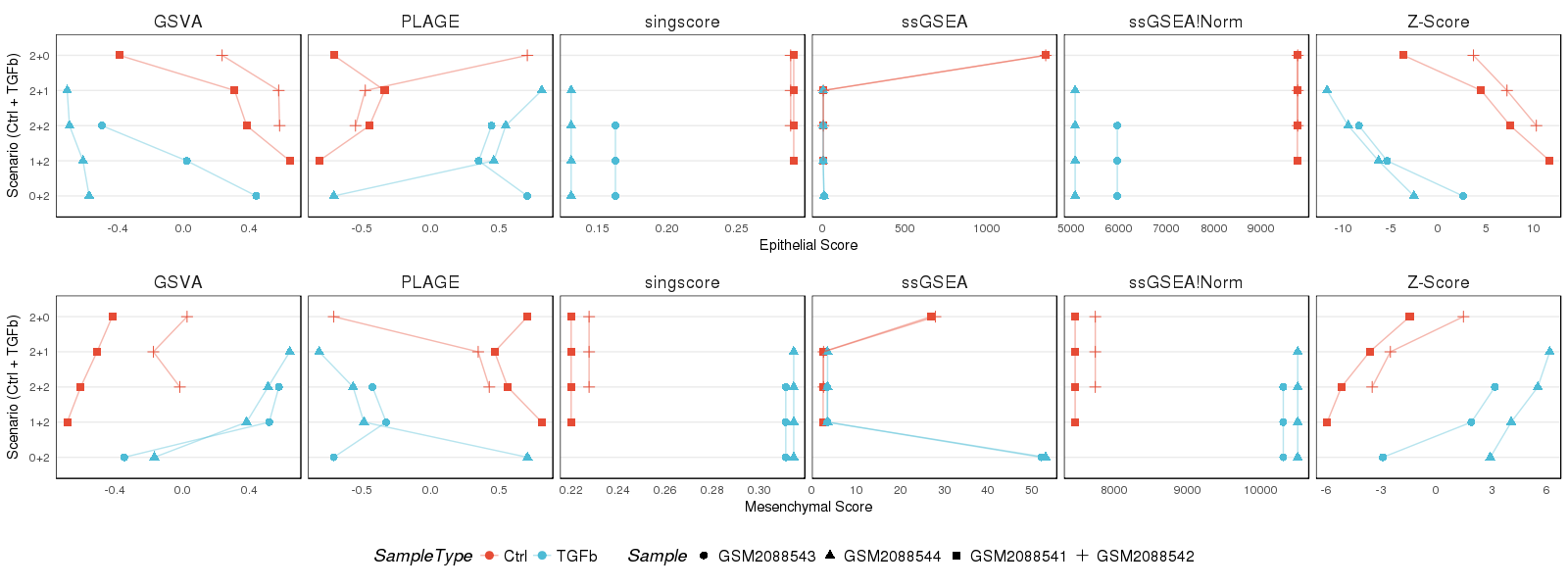


**Figure S7.** Comparing the stability of scores obtained from different methods for a small number of cell line samples (data from GSE79235; 2 control and 2 TGFβ-treated samples).

# Compute time for different scoring methods

We compared the compute time required for each scoring method across 25 or 500 samples from the TCGA breast cancer RNASeq data, and 10000 randomly-selected signatures from MSigDB (each re-sampled 20 times).


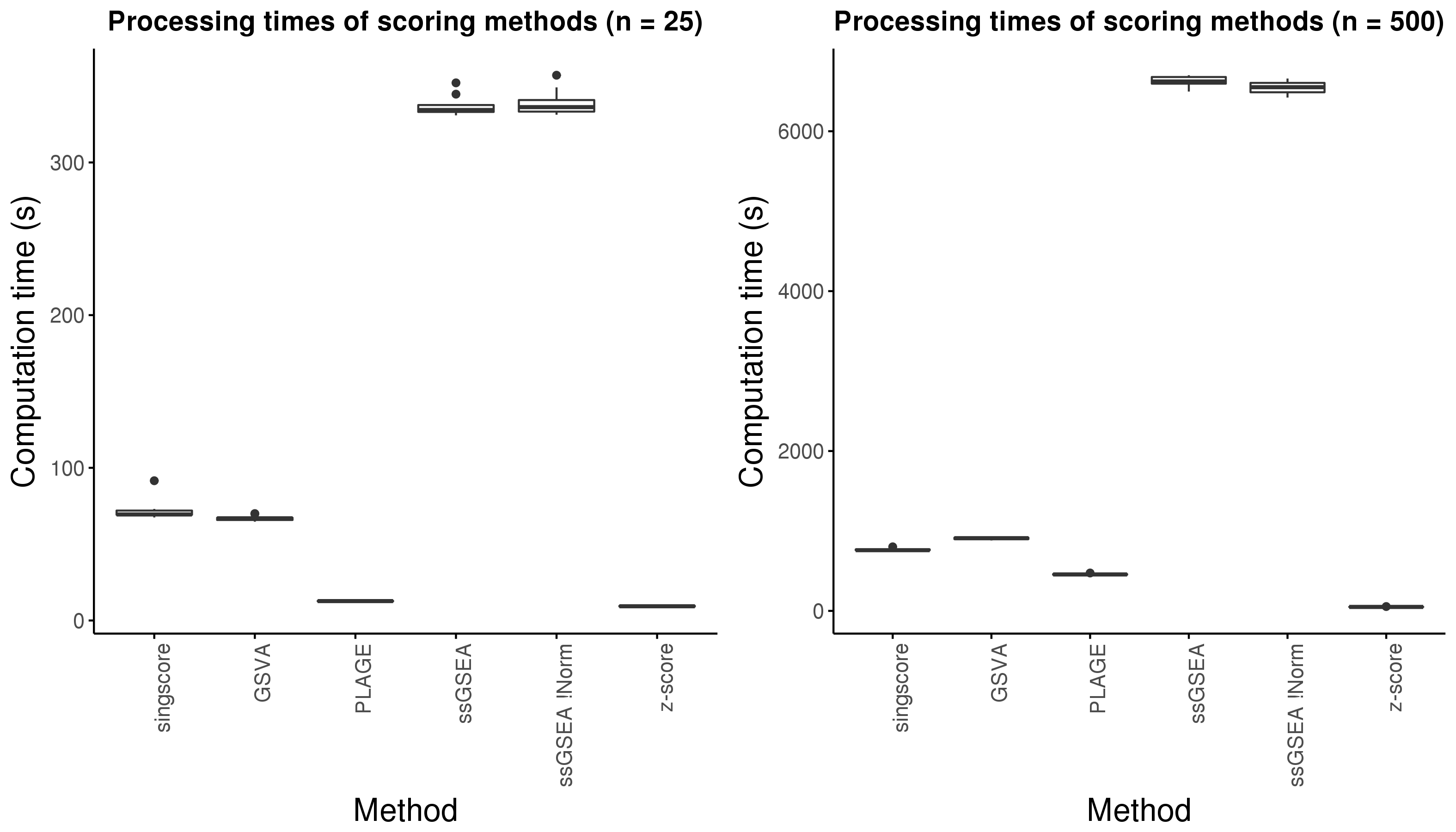


**Figure S8.** Comparing the compute times for each scoring method (*singscore*, GSVA, PLAGE, ssGSEA, ssGSEA_!Norm_, and z‑score) in two groups sub-sampled from the TCGA breast cancer RNA-seq data (*N_S_* = 25 and *N_S_* = 500). These comparisons were performed on a UNIX machine (Intel(R) Xeon ® CPU E5-2690 v3 @ 2.60GHz) without code parallelisation.
